# On the basic mechanisms of anticipated synchronization in neuronal circuits

**DOI:** 10.1186/1471-2202-16-S1-P167

**Published:** 2015-12-18

**Authors:** Fernanda Matias, Ana Paula Millan, Luis Martinez, Santiago Canals, Pedro Carelli, Mauro Copelli, Claudio R Mirasso

**Affiliations:** 1 Instituto de Física, Universidade Federal de Alagoas, 57072-900 Maceió, Brazil; 2 Departamento de Electromagnetismo y Física de la Materia, Universidad de Granada, 18071 Granada, Spain; 3 Instituto de Nuerociencias de Alicante, 03690 Sant Joan d'Alacant, Spain; 4 Departamento de Física, Universidade Federal de Pernambuco, Recife PE 50670-901, Brazil; 5 Instituto de Fisica Interdisciplinar y Sistemas Complejos, CSIC-UIB, Campus Universitat de les Illes Balears, E-07122 Palma de Mallorca, Spain

## 

Anticipated synchronization (AS) is an anti-intuitive phenomenon that can occur in two coupled dynamical systems when there is a dominant connectivity between the elements. AS occurs when a dynamical system A dominantly connects to another system B and B synchronously pulses before A does. It has been recently shown [1,2] that AS can occur in a model of coupled Hodgkin-Huxley (HH) neurons and even in neuron populations. Recently, this astonishing regime has been observed in some cortical circuits of monkeys when performing a visual discrimination task [2]. However, the basic mechanisms for this synchronization to occur are still unclear. In this communication we analyze a circuit of excitatory and inhibitory HH neurons as well as neurons populations and find, analyzing individual responses as well as phase response curves, that inhibitory neurons can control the transition between delayed and anticipated synchronization.
